# Epitope reactions can be gauged by relative antibody discriminating specificity (RADS) values supported by deletion, substitution and cysteine bridge formation analyses: potential uses in pathogenesis studies

**DOI:** 10.1186/1756-0500-5-208

**Published:** 2012-07-05

**Authors:** Andrew K I Falconar

**Affiliations:** 1 Laboratorio de Investigaciones en Enfermedades Tropicales, Departamento de Medicina, Universidad del Norte, Km5 Antigua via Puerto Colombia, Barranquilla, Colombia; 2 Department of Pathogen Molecular Biology, London School of Hygiene and Tropical Medicine, Keppel Street, London, WC1E 7HT, United Kingdom

**Keywords:** Epitope, Mapping, Synthetic peptide, Monoclonal antibody, Relative antibody discriminating specificity value

## Abstract

**Background:**

Epitope-mapping of infectious agents is essential for pathogenesis studies. Since polyclonal antibodies (PAbs) and monoclonal antibodies (MAbs) are always polyspecific and can react with multiple epitopes, it is important to distinguish between specific and non-specific reactions. Relative antibody discriminating specificity (RADS) values, obtained from their relative ELISA reactions with L-amino acid peptides prepared in the natural versus reverse orientations (x-fold absorbance natural/absorbance reverse = RADS value) may be valuable for this purpose.

PAbs generated against the dengue type-2 virus (DENV-2) nonstructural-1 (NS1) glycoprotein candidate vaccine also reacted with both DENV envelope (E) glycoproteins and blood-clotting proteins. New xKGSx/xSGKx amino acid motifs were identified on DENV-2 glycoproteins, HIV-1 gp41 and factor IXa. Their potential roles in DENV and HIV-1 antibody-enhanced replication (AER) and auto-immunity were assessed.

In this study, a) RADS values were determined for MAbs and PAbs, generated in congeneic (H2: class II) mice against DENV NS1 glycoprotein epitopes, to account for their cross-reaction patterns, and b) MAb 1G5.3 reactions with xKGSx/xSGKx motifs present in the DENV-4 NS1, E and HIV-1 glycoproteins and factor IXa were assessed after the introduction of amino acid substitutions, deletions, or intra-/inter-cysteine (C-C) bridges.

**Results:**

MAbs 1H7.4, 5H4.3, 3D1.4 and 1G5.3 had high (4.23- to 16.83-fold) RADS values against single epitopes on the DENV-2 NS1 glycoprotein, and MAb 3D1.4 defined the DENV complex-conserved LX1 epitope. In contrast, MAbs 1G5.4-A1-C3 and 1C6.3 had low (0.47- to 1.67-fold) RADS values against multiple epitopes. PAb DENV complex-reactions occurred through moderately-high (2.77- and 3.11-fold) RADS values against the LX1 epitope. MAb 1G5.3 reacted with xSGKx motifs present in DENV-4 NS1 and E glycoproteins, HIV-1 gp41 and factor IXa, while natural C-C bridge formations or certain amino acid substitutions increased its binding activity.

**Conclusions:**

These results: i) were readily obtained using a standard 96-well ELISA format, ii) showed the LX1 epitope to be the immuno-dominant DENV complex determinant in the NS1 glycoprotein, iii) supported an antigenic co-evolution of the DENV NS1 and E glycoproteins, and iv) identified methods that made it possible to determine the role of anti-DENV PAb reactions in viral pathogenesis.

## Background

Synthetic peptides, covalently attached to solid-phase supports or displayed on phage particles so that each amino acid side chain accessible to MAb or PAb binding are powerful tools to precisely locate epitopes on a wide range of viral proteins for studying pathogenicity, and accessing potential vaccines and immunotherapies [1]. The rapid preparation of thousands of synthetic peptides, covalently coupled by their carboxyl-termini, at uniform concentrations (Chiron Mimetopes, UK) has greatly facilitated such studies using the standard 96-well ELISA format [2-4]. This method gave highly reproducible very consistent results when numerous research groups (n = 16) used it for B-cell epitope-mapping [5], and the results obtained with duplicate peptides were very similar (Absorbance (Abs) ranges: 0.00 to 0.06) [6-8]. This method avoided the problems encountered when short peptides were adsorbed to solid-phase supports, and was useful for studying the effects of inter- and intra-cysteine bridge formation that occur in many B-cell epitopes. When the three-dimensional structure of the antigen is known, it is possible to select optimal peptide sequences for testing the binding activity of MAbs and PAbs.

PAbs and MAbs, as well as T-cells, are able to react with numerous epitopes that present little similarity in amino acid sequence and which may also be present on auto-antigens (host proteins) [9-14]. These ‘cross-reactions’ gave rise to polyspecificity which has been described to epitope ‘degeneracy’, ‘plasticity’ or to ‘molecular mimicry’ [15]. It has been suggested that ‘poly-functional’ antibodies (i.e. those able to bind to multiple epitopes) could be an advantage for the host and account for promoting antibody maturation [10], although such T-cell and B-cell reactions with epitopes in host proteins could result in autoimmune disease. Despite considerable debate, there is no single accepted theory to account for the acquired immune system’s ability to avoid what was originally termed ‘*Horror Autotoxicosis*’ [13,16-18]. A number of factors are believed to contribute to antigenic ‘polyspecificity (cross-reactions) such as hydropathic complementarity [19,20] and epitope length, since long peptides which contained identical tri- and tetra- amino acid sequences was sufficient to result in cross-reactions [21]. It has been extremely difficult to design vaccine candidates by the reconstruction of native structures of viral neutralization epitopes using synthetic peptides [14,22]. Synthetic peptides have however been very useful for identifying many epitopes that were common to the dengue virus (DENV) nonstructural-1 (NS1) glycoproteins and proteins from human thrombocytes, endothelial cells and blood-clotting factors [6,23]. Since the DENV NS1 glycoprotein is a candidate vaccine, it is essential to fully evaluate the role of these PAb cross-reactions in pathogenesis by performing these experiments *in vivo* under normal physiological conditions [23,24].

The dengue viruses (DENVs) are important pathogens of humans and, since they exist as four discrete serotypes, they may cause four sequential infections in many countries where all four DENV serotypes co-circulate [25]. The DENVs are immunologically interesting due to evidence of strain variation in their pathogenic capacities, and because PAbs generated against one DENV serotype are able to increase the replication of heterologous DENV serotypes in Fcγ receptor-bearing monocytes/macrophages using either PAbs or MAbs *in vitro* or *in vivo*[26,27]. While this phenomenon is generally termed ‘antibody-dependent enhancement’ (ADE), an alternative term ‘antibody-enhanced replication’ (AER) was introduced to prevent confusion with antibody-enhanced disease (AED) that occurred in the absence of ADE [7,24]. The most severe and life-threatening DENV disease, dengue haemorrhagic fever and dengue shock syndrome (DHF/DSS) usually results from secondary DENV infections [26-28], particularly by DENV-2 and DENV-4 [28]. These observations were supported by finding that the 131-HH versus the 131-RR genetic polymorphism in the FcγRIIa, which significantly increased the binding of immune complexes containing the human IgG1, IgG2 or IgG3 subclasses [29], which generated increased DENV AER, and was therefore a significant DHF/DSS risk factor [30,31].

The generation of DENV-2 AER *in vitro* resulted in the down-regulation of type-I interferon and *RIG-I/MDA5*-signalling pathways and pro-inflammatory genes, thereby allowing increased DENV replication to occur *in vitro*[27,32-34]. These innate immune system genes were also down-regulated in the acute-phase blood samples from DHF/DSS patients [27,35,36], which further indicated that DENV AER plays a key role in DHF/DSS pathogenesis. Of particular concern for DENV vaccine development is the fact that sub-neutralizing concentrations of all PAbs generated against any DENV serotype may result in their AER [26]. Any candidate DENV vaccine must therefore generate appropriate, and life-long sustainable, neutralizing PAb titers against each DENV serotype [37,38].

As a potential way to generate protective PAbs against all four DENV serotypes, without the risk of DENV AER, the DENV nonstructural-1 (NS1) glycoprotein was proposed as a suitable candidate subunit vaccine [39,40]. The DENV NS1 glycoprotein is however released as a homo-hexameric glycoprotein at high concentrations from DENV-infected cells con-currently with DENV virions. IgG responses against these glycoproteins are only observed during the acute-phase of secondary DENV infections [41], when most DHF/DSS cases occur [26,28], and common epitopes were identified on the DENV-2 NS1 and E glycoproteins [7].

Despite possessing RNA genomes, phylogenetic analyses of the DENV-2 strains using their E glycoprotein-encoding genes, together with antigenic analysis of their NS1 glycoproteins using MAbs, suggested that the DENV-2 E and NS1 glycoproteins co-evolved [42]. These results were further supported by phylogenetic analyses of the E and NS1 glycoprotein-encoding genes of all four DENV serotypes [43]. It was therefore hypothesized that common epitopes on the E and NS1 glycoproteins could influence DENV AER by either NS1 glycoproteins acting as ‘immunological decoys’, or by the cross-reactions of PAbs generated against the DENV NS1 glycoproteins with their E glycoproteins [7]. This theory was recently supported by the demonstration that undiluted PAbs, raised against the DENV-2 NS1 glycoprotein in normal out-bred mice, also reacted with epitopes on the DENV-2 E glycoprotein, and generated a dramatic AER of a wild-type DENV-2 strain which resulted in lethal multi-organ disease syndrome (MODS), similar to that reported in DHF/DSS patients [24].

In contrast to single epitopes which were defined by some MAbs on the DENV-2 NS1 glycoprotein [44], PAbs and some MAbs were also generated against defined short 3-4-mer basic (K, R or sometimes H)-and/-or acidic (E or D)-hydrophobic (usually L, I, or V)-basic (K, R or sometimes H) amino acid motifs present in either the natural or reverse orientations, collectively termed ELK/KLE-type and KELK/KLEK-type motifs [6,23]. These ELK/KLE-type motifs were i) immuno-dominant in all congeneic (class II) mouse tested, as well as in humans [7], ii) more strongly detected by PAbs from DSS than DF patients, iii) more strongly detected throughout the protein by both PAbs from DSS patients and high responding (H-2^b^ and H2^s^) mouse haplotypes, and by MAb 1G5.4-A1-C3 than by its low affinity sister clone, 1G5.4-A1-H6 [6,23], and iv) also more numerous and/or antigenic in surface-exposed regions of the DENV E glycoproteins from virulent (DHF/DSS-associated) DENV-2 and DENV-3 strains [7], strongly suggesting that they may have generated the DENV-2 AER/AED observed using undiluted anti-DENV NS1 glycoprotein sera [24]. These results also agreed with the findings that: i) all of these PAbs cross-reacted with critical sites in human blood-clotting factors (e.g. fibrinogen and factor VII), platelets and endothelial cells [6,23], ii) fibrinogen was the dominant auto-antigen identified in DSS patients’ circulating immune complexes [45], iii) epitope density (numbers of similar exposed determinants/protein) played a key role in immunogenicity and antigenicity [46-49] and iv) in multiple infectious diseases such immunodominant repetitive determinants diverted the hosts’ immune responses [50-56]. It seems likely therefore that repetitive motifs in DENV proteins such as the ELK/KLE-type motifs, which were more numerous and/or antigenic in the main envelope (E) glycoproteins of virulent (DHF/DSS-associated) DENV strains [7], play an important role in the pathogenesis of DHF/DSS through increased AER/AED.

In addition, to the ELK/KLE-type and KELK/KLEK-type motifs, other short (e.g. SGK/KGS-type) motifs were also identified on the DENV NS1 glycoproteins. These sites contained degenerate amino-acids in their flanking xSGKx/xKGSx regions, whilst the core SGK sequence was maintained. The xKGSx motif was identified in a neutralizing epitope on the E glycoproteins of all DENV serotypes [8], while xSGKx motifs were uniquely located in exposed neutralizing regions of domain II and III of the DENV-4 E glycoprotein. The xSGKx motif was also identified within an immuno-dominant epitope (600-GIWGCSGKLICTTA-612 on the glycoprotein-41 (gp41) of HIV-1 [57,58]. Stronger reactions against this epitope were observed using PAbs from HIV-1-infected AIDS versus non-AIDS patients, and these PAbs generated HIV-1 AER via complement receptors [59]. In one study, however, a human MAb that defined this epitope also generated HIV-1 AER via a complement-independent, Fcγ receptor-mediated mechanism [60]. The xSGKx motif was also identified in the critical phospho-lipid cell-binding site (1-YNSGKLEEFV-10) in the conformationally-dependent ω-loop of the Gla domain on activated mammalian vitamin K-dependent blood-clotting factor IX [61].

PAbs generated by congeneic (class II) mouse strains against the DENV-2 NS1 glycoprotein showed different (DENV-2 type, DENV-2/-4 sub-complex and DENV-1 to −4 complex) reaction patterns with the NS1 glycoproteins of each DENV serotype [6,23]. The relative ability of MAbs and these PAbs to react with all of the known sequential epitopes on the DENV-2 NS1 glycoprotein, when they were prepared in the natural and reverse orientations (relative antibody discriminating specificity (RADS) (absorbance natural/absorbance reverse = x-fold) values), were hypothesized to discriminate between specific and non-specific MAb and PAb reactions. In this case, these RADS values may be very useful for identifying MAb and PAb reactions with the DENV E glycoproteins or host auto-antigens, which could generate DENV AER and/or auto-immunity.

This is therefore the first study attempting to: A) determine the RADS values of MAbs that defined single or multiple epitopes on DENV NS1 glycoproteins, B) determine and compare the RADS values of the PAbs generated against the DENV-2 NS1 glycoprotein by different congeneic (H-2 class II) mouse strains with MAb RADS values, to account for their reaction (DENV-2-type, sub-complex or complex) profiles against the NS1 glycoproteins of each DENV serotype, C) determine the RADS values of MAb 1G5.3 against its DENV-2 NS1 glycoprotein target 24C epitope, and the xKGSx motif present in a neutralizing epitope on the E glycoproteins of all four DENV serotypes, D) assess the ability of MAb 1G5.3 to react with the xSGKx motifs in: i) DENV-4 NS1 and E glycoproteins, ii) an immuno-dominant HIV-1 gp41 epitope, and iii) a critical platelet-binding sequence in the activated mammalian blood-clotting factor IXa, when these epitopes contained intra-/inter-cysteine-bridges, amino acid deletions or substitutions and more closely mimicked their native protein conformations. Where possible, these reactions were shown to be compatible with the location of the epitopes in the three-dimensional structure of the protein.

The present study aimed to establish the value of this new methodology for measuring: a) epitope reactions when better peptide mimics were designed and tested, b) the capacities of PAbs to generate AER of DENVs of each serotype or of other infectious agents (e.g. HIV-1), and c) the capacities of PAbs to cross-react with host proteins (auto-antigens), that may be implicated in DHF/DSS pathogenesis (through vascular leakage and/or haemorrhage), or in other infectious and non-infectious diseases.

## Methods

### Antigen preparation and purification

The growth of dengue viruses in mammalian cells and the preparation of virus-infected cell sodium dodecyl sulphate (SDS) lysates for Western blotting assays were as described [7,23,62]. Briefly, dengue viruses of each serotype (DENV-1 (Nauru Island strain), DENV-2 (PR159 and TR1751 strains), DENV-3 (H87 strain) and DENV-4 (H-241 strain) were used to infect 70% confluent Vero cell monolayers maintained in medium 199 with 4% (vol/vol) foetal calf serum and antibiotics. After incubation at 37°C for 5 days, the supernatants were collected, the cell monolayers washed with medium 199, and the infected cells homogenized using 0.5% (wt/vol) sodium dodecyl sulphate (SDS) in 330 mM phosphoric acid and 0.71% (wt/vol) Trisma base (pH 6.8). Aliquots were stored at −80°C.

The immuno-affinity purification of the native homo-hexameric extracellular/secreted (e/s) form of the NS1 (e/sNS1) glycoprotein was as described [6,7,23]. Briefly, Vero cell monolayers (10 × 225 cm^2^ flasks) were infected with DENV-2 (PR 159 strain), the supernatants were harvested on days 5 and 8 after infection, and: i) clarified by centrifugation, ii) sodium azide and a cocktail of protease inhibitors were added, iii) they were slowly passed through an immuno-affinity column containing 12 mg of MAb 2A5.1 covalently coupled to Sepharose 4B, iv) the column was washed with PBS containing protease inhibitors, v) the bound DENV-2 e/sNS1 glycoprotein was eluted in the native form using 20 mM diethylamine in PBS containing protease inhibitors (pH 11.2), and vi) 500 μl fractions were immediately neutralized with 100 μl of 1 M Tris/HCl pH 7.2. Protein concentrations were determined using an ELISA plate-adapted bichinchonic acid (BCA) protein determination assay (Pierce, USA) using standard 10 to 1,000 μg/ml) bovine serum albumin concentrations, and Western blotting analyses of each protein-containing fraction were performed using MAb 1A12.3 as described [7,23,62].

### Preparation and purification of mouse monoclonal antibodies (MAbs)

The preparation of 173 MAbs against the native homo-hexameric e/sNS1 form of DENV-2 (PR159 strain), and the further characterization of MAbs 1H7.4, 5H4.3, 3D1.4, 1G5.3, 1C6.3 and 1G5.4-A1-C3, were previously described [6,7,23,62]. Each of these MAbs reacted with the native hexameric e/sNS1 form of the protein in ELISA, and both the dimeric and monomeric forms of these proteins in Western blot assays. These MAbs were purified by binding to protein G-Sepharose 4B fast flow (P-3296: Sigma, USA) using 0.1 M Na_2_HPO_4_ containing 300 mM NaCl (pH 7.2), eluted using 0.1 M glycine/HCl (pH 2.7), and 1 ml fractions were immediately neutralized with 100 μl of 1.5 M Tris/HCl (pH 7.8). The MAb concentrations in each fraction were then determined using the BCA assay (see above).

### Polyclonal antibody (PAb) production

All of the studies using mice were performed under a personal and project animal (scientific procedures) licences (PIL 70/6903) issued by the Home Office, UK. The immunization of congeneic mice with the native purified hexameric DENV-2 e/sNS1 glycoprotein was as described [6,23]. Briefly, groups of three 3-week old mice of the B10.S, B10.RIII, B10.G, B10.BR, B10.A, B10.D2N and C57BL/BJ strains were immunized using the combined intra-peritoneal/subcutaneous (i.p/s.c.) routes with 10 μg of the purified DENV-2 (TR1751 strain) e/sNS1 glycoprotein emulsified in Freund's complete adjuvant, boosted with the same antigen dose contained in PBS two weeks later. Blood from each animal was collected a further two weeks later, and their separated sera stored at −80°C. A group of the high-responding B10.S strain mice were immunized with 10 μg doses of ovalbumin (A-7641 Sigma, USA) as controls using the same methodology.

### Peptide synthesis

The preparation and use of synthetic peptides, covalently attached to polypropylene ‘pins’, ‘gears’ and ‘arrowheads' (Chiron Mimetopes, United Kingdom), has been described [6-8,23,44,62]. Briefly, duplicate synthetic peptide sequences of epitopes identified by MAbs 1H7.4, 5H4.3, 3D1.4, 1G5.3, 1C6.3 and 1G5.4-A1-C3 on the DENV-2 NS1 glycoprotein were prepared using activated Fmoc-protected L-amino acid esters (Novabiochem, United Kingdom) in both natural and reverse (unnatural) orientations on ‘gears/arrowheads’. The amino-terminus of each peptide was then acetylated and the side-chain protective groups removed using a 91.3% (wt/vol) trifluoroacetic acid (TFA) mixture. Peptide alterations were prepared containing amino acid inversions, deletions, substitutions and intra-/inter-cysteine (C) bridge (C-C) formations to test the reactions of MAb 1G5.3. The surface-exposed target sequences on the DENV-4 E glycoprotein were identified and confirmed by comparisons with the corresponding sequences in high-resolution x-ray crystallographic determinations of the dimeric forms of the DENV-2 and DENV-3 E glycoproteins (http://www.ncbi.nlm.nih.gov**MMBD ID 23080** and **32273]**) [63,64]. Examples for MAb reaction studies with epitopes on other viruses or human proteins were chosen since; a) a full analysis of the optimal peptide length and need for the intra-cysteine bridge in the immuno-dominant epitope on gp41 of HIV-1 had been performed [57,58], b) high resolution X-ray crystallographic data had been determined for the 600-IWGCSGKLICTTA-612 epitope on HIV-1 gp41 **[MMDB ID 73687]**[65] and c) high-resolution x-ray crystallographic data were available for the mammalian-conserved, and Ca^2+^-stabilised/activated, γ-carboxyglutamic acid- (γE)-rich (Gla domain (ω-loop)) epitope on blood-clotting factor IXa (1-YNSGKLγEγEFV-10), bound by either a MAb 10C12 (Fab fragment **MMDB ID 25991]**[66] or the snake (*Trimeresurus flavoviridis*) venom protein, (IX/X-bp) **MMDB ID 23297** and **23298]**[67]. This region was essential for the binding of factor IXa to platelet phosphatidylserine-containing phospholipid membranes, which act as a stage for its subsequent activation of blood-clotting factor X, but which was inhibited by both MAb 10C12 and IX/X-bp [61,66,67].

Intra-peptide cysteine (C-C) bridges were prepared using Fmoc-C(Trt) (N.B. Trt group was removed during TFA treatment) for both cysteine (C) residues in the peptides, with subsequent oxidation in air-saturated distilled water adjusted to pH 8.0 with 0.01 M NH_4_OH for 8 hours. In contrast, inter-peptide cysteine (C-C) bridges were prepared by using Fmoc-C(Trt) for one of the C residues and Fmoc-C(*S*-Acm) for the second C residue. The first inter-C-C bond was formed by air oxidation (see above), while the second inter-C-C bond was formed by incubation of the peptides in 2 μM I_2_ in 20% (vol/vol) methanol for 1 hour.

The RLX1 peptide sequence (110-LRYSWKTWGKAKMLSTEL-127C) differed from the KLX1 peptide described previously [6,23] by containing the potentially more antigenic arginine (R) residue at position 111 (underlined). This sequence was present in the DENV-2 NS1 glycoproteins of the PR159 and TR1751 strains used to prepare the MAbs and immunize the congeneic mouse strains, respectively. This peptide was prepared on 100–200 mesh Cys(TrT)-Wang resin using HBTU/activated Fmoc-protected amino acids on a multiple peptide synthesizer (Zinsser Analytic SMPS350: Zinsser, United Kingdom), and cleaved with a 91.3% (wt/vol) trifluoroacetic acid mixture. After being repeatedly precipitated in cold diethyl ether, the peptide was dried under argon. The additional Boc groups were removed from the tryptophan (W) residues by incubation with 20% (vol/vol) glacial acetic acid/H_2_O for four hours at 25°C. This mixture was then made to 5% (vol/vol) acetonitrile, loaded onto a preparative C18 reversed-phase column (Vydac, USA) and subjected to high performance liquid chromatography (Beckman Gold, USA) using a 5–95% (vol/vol) acetonitrile gradient containing 1% (wt/vol) TFA (pH 2.0), and the main peak was collected and freeze-dried.

### Immunoassays

The indirect ELISAs, using purified hexameric e/sNS1 glycoprotein and synthetic peptides, have been described [6,7,23,24,44]. For these assays, 96-well ELISA plates were loaded with the purified DENV-2 e/sNS1 glycoprotein or the RLX1 peptide at 10 μg/ml (50 μl/well) diluted in carbonate/bicarbonate buffer (pH 9.8) overnight at 4°C. After washing with PBS containing 0.05% (vol/vol) Tween20 (PBS/T), they were blocked using 1% (wt/vol) gelatin/PBS for 2 hours at 25°C, and after washing with PBS/T, duplicate serial 3-4-fold dilutions of purified mouse MAbs or PAbs from 1/20 were prepared in PBS/T containing 0.25% (wt/vol) gelatin (PBS/T/G) and allowed to react 37°C for 60 minutes. After washing with PBS/T, the bound mouse antibodies were detected by sequential steps involving peroxidase-labeled goat anti-mouse IgG (H&L) (115-035-166: Jackson ImmunoResearch, USA) in PBS/T/G, washing with PBS/T, incubation with *o*-phenylene diamine (opd) in citrate/phosphate buffer (pH 5.0) containing H_2_O_2_, and the absorbance values measured at 490 nm (MRX: Dynex, USA). The results were expressed as the average reciprocal log_10_ 50% end-point titres (1/log_10_t_50_). For the peptide-coated ‘gears/arrowheads’, 175 μl volumes of PBS/G were used for the blocking, and the purified MAbs or pooled PAbs collected from each congeneic mouse strain were reacted at 50 times their average 1/log_10_t_50_ ELISA titres obtained against the purified hexameric DENV-2 e/sNS1 glycoprotein in 96-well ELISA plates (150 μl/well). All of the other steps were also performed using 150 μl volumes and the average absorbance values were determined at 490 nm. These peptide coated gears/arrowheads were then recycled as described previously [6,7,23].

Non-reduced immunoblotting (Western blotting) was performed using the DENV-infected cell lysates or purified DENV-2 e/sNS1 glycoprotein as described previously [6,7,23,24]. Briefly, serial 8.0-0.5 μl sample dilutions of each of these lysates, or 100 ng of the DENV-2 e/sNS1 glycoprotein, were: i) made to 10 μl in 1x stacking buffer containing 0.5% (wt/vol) SDS, ii) heated at 100°C for 3 minutes, iii) subjected to electrophoresis (15–20 constant mA/gel) on a 7% (wt/vol) SDS-PAGE system (Miniprotean II: BioRad, UK), iv) electro-blotted at 160 mA/gel (Sartoblot II, Sartorius, UK) onto 0.2 μm pore-size nitrocellulose membranes, and v) air-dried. These membranes were then blocked with 3% (wt/vol) skimmed milk powder/PBS (PBS/T/M) containing 0.02% (wt/vol) NaN_3_ overnight. The DENV-complex-reactive MAb 1A12.3 (2.5 μg/ml) was then reacted with these membranes for 2 hours at 25°C. After washing with PBS/T (4 × 5 minutes), the bound MAbs were detected using sequential steps of washing with PBS/T, peroxidase-labelled goat anti-mouse IgG (H&L) (see above), and 4-chloro-1-naphthol/3,3’diaminobenzidine 4HCl substrate in PBS containing H_2_O_2_. The reaction intensities of this MAb was then used to adjust the concentrations of the NS1 glycoproteins in the infected-cell lysates of each DENV serotype to gauge the immunoblot cross-reactive intensities of the other MAbs and PAbs generated against these glycoproteins.

## Results

### MAb and PAb ELISA and immunoblot reactions against DENV NS1 glycoproteins and the RLX1 peptide

MAbs 1H7.4 (DENV-2 type-reactive: LD2 epitope), 5H4.3 (DENV-2/-4 sub-complex-reactive: 24A epitope), 3D1.4 (DENV-1 to −4 complex-reactive: LX1 epitope-specific) and 1G5.3 (DENV-2/-4 sub-complex-reactive: 24C epitope-specific), which defined single sequential epitopes on the DENV-2 NS1 glycoprotein [6,7,44], strongly reacted with DENV-2 NS1 glycoprotein in the ELISA (Table 1). Only MAb 3D1.4 strongly reacted with the RLX1 peptide sequence (110-LRYSWKTWGKAKMLSTEL-127C), which contained the antigenically-conserved DENV complex LX1 epitope (underlined). In contrast, MAbs 1C6.3 and 1G5.4-A1-C3 defined multiple KELK- (basic-acidic-hydrophobic-basic amino-acid) and ELK- (acidic-hydrophobic-basic amino-acid) type motifs on DENV NS1 glycoproteins that were naturally present in either orientations (e.g. KELK/KLEK and ELK/KLE).

**Table 1:** MAb/PAb reactions against DENV NS1 glycoproteins and the RLX1 peptide

**Antibody**^**a**^	**Epitope (motif)**^**b**^	**1/log**_**10**_**50% end-point ELISA titre**^**c**^		**Immunoblot reactions against non-reduced NS1 glycoproteins**^**d**^			
		**D2V NS1 glycoprotein**	**RLX1 peptide**	**D1V**	**D2V**	**D3V**	**D4V**
**MAb**							
1H7.4	LD2	6.0	<1.5	++++	++++		
5H4.3	24A	5.2	<1.5	+++	+++	++	++
3D1.4	LX1	5.4	4.7	+++	+++	+++	+++
1G5.3	24C	5.7	<1.5	+++	+++	+	+++
1C6.3	(KELK/KLEK)	4.5	1.7	+++	++	++	+++
1G5.4-A1-C3	(ELK/KLE)	5.2	1.9	++	++++	+	+++
**PAb (Haplotype)**							
C57BL/BJ (I-A^**b**^)	Multiple	4.5	2.9	+++	+++	++	+++
B10.S (I-A^**s**^)	Multiple	4.7	3.0	++	+++	++	+++
B10.RIII (I-A^**r**^*,*I-E^**r**^)	Multiple	3.8	2.2	+	++	+	+
B10.BR (I-A^**k**^,I-A^**k**^)	Multiple	3.7	2.0	++	++	+	+
B10.G (I-A^**q**^)	Multiple	4.0	2.1	++	++		
B10.D2N (I-A^**d**^,I-E^**d**^)	Multiple	4.0	2.1	+	+		+
B10.A (I-A^**k**^)	Multiple	3.5	1.7	+	+		
B10.S (C) I-A^**s**^)	Multiple	<1.0	<1.5				

^a^Monoclonal antibodies (MAbs) or polyclonal antibodies (PAbs) generated against the native hexameric.

DENV-2 NS1 glycoprotein or control protein (ovalbumin) (B10.S (C)) in congeneic mouse strains with the H2 class II (I-E and/or I-A molecule) haplotypes shown in brackets.

^b^Epitope name or reaction with multiple epitopes (PAbs), or MAbs which defined multiple ELK/KLE-type and KELK/KLEK-type motifs in multiple peptide sequences from the DENV-2 NS1 glycoprotein.

^c^Reciprocal log_10_ 50% end-point ELISA titers against immuno-affinity purified native hexameric DENV-2 (D2V) s/eNS1 glycoprotein and the RLX1 synthetic peptide.

^d^Immunoblot colour reaction intensity gauged on an arbitrary scale from negative (blank) to ++++ against the non-reduced NS1 glycoproteins of DENV-1 (D1V) to DENV-4 (D4V).

MAb 3D1.4 displayed similar reaction intensities against the NS1 glycoprotein of each DENV serotype, while MAb 1C6.3 displayed weaker reactions against the DENV-2 and DENV-3 NS1 glycoproteins, and MAb 1G5.4-A1-C3 showed a DENV-2 > DENV-4 > DENV-1 > DENV-3 reaction profile in the immuno-blot assays.

Congeneic (H-2 class II: I-A ± I-E) mouse strains, immunized with the native form of the DENV-2 NS1 glycoprotein, all generated moderate to high PAb titres against this homologous glycoprotein in the ELISA. The B10.A (I-A^k^), B10.BR (I-A^k^,I-E^k^) and B10.RIII (I-A^r^,I-E^r^) mouse strains possessed relatively low-responding class II haplotypes (average 1/log_10_t_50_ ELISA titers ≤ 3.8), while C57BL (I-A^b^) and B10.S (I-A^s^) mice were identified as possessing high-responding class II haplotypes (average 1/log_10_t_50_ ELISA titers ≥ 4.5). Only the PAbs generated by these high-responding (C57BL and B10.S) mouse strains strongly reacted with the RLX1 peptide and the NS1 glycoprotein of all four DENV serotypes. These two high-responding mouse strains therefore, uniquely, appeared to generate PAbs against the LX1 epitope. In contrast, the control B10.S (H2 I-A^s^) mouse PAbs generated against ovalbumin (OA) failed to react with either the DENV NS1 glycoproteins or the RLX1 peptide.

### MAb reactions against single-target epitopes prepared in the natural and reverse orientations using L-amino acids

**Figure 1:**
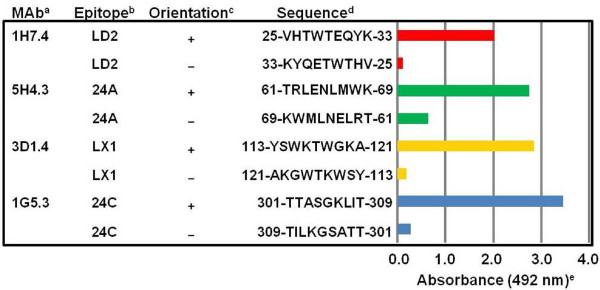
**MAb reactions against single-target DENV-2 NS1 glycoprotein peptide sequences prepared in natural and reverse orientations.**^a^Monoclonal antibody (MAb) name. ^b^Epitope name on the DENV-2 NS1 glycoprotein. ^c^Epitope sequence prepared in the natural (+) or reverse (−) orientations. ^d^Amino acid sequence position and single letter amino acid peptide sequence. ^e^Average ELISA absorbance (490 nm) against each duplicate peptide when the MAbs were diluted at 50 times their 50% end-point ELISA titers obtained against the native hexameric DENV-2 e/sNS1 glycoprotein (standard deviation range, 0.00 (low values) to 0.07 (high values)).

The four MAbs, which defined single sequential epitopes on the DENV-2 NS1 glycoprotein, reacted strongly with their single-target epitope sequences when they were prepared in the natural (+) orientation (Figure 1). MAb 1H7.4, which defined epitope LD2 (25-VHTWTEQYK-33), had a very high 16.83-fold relative antibody discriminating specificity (RADS) value, obtained from the comparison of its absorbance (Abs) against the natural/reverse orientated target peptide sequences (Abs natural (+) 2.02/Abs reverse (−) 0.12 = 16.83-fold RADS value) (Figure 1, Table 2, Table 3). High RADS values were also obtained for the other three MAbs. MAb 3D1.4 had a 15.00-fold RADS value against epitope LX1, MAb 1G5.3 had 12.36-fold RADS value against epitope 24C, and MAb 5H4.3 had a 4.23-fold RADS value against epitope 24A. These four MAbs therefore had moderately-high abilities to discriminate between their natural and reverse orientated synthetic peptide target sequences.

**Table 2:** Mouse PAb reactions against DENV-2 NS1 glycoprotein peptides prepared in the natural and reverse orientations

**Peptide/Epitope**^**a**^	**N/R**^**b**^	**Sequence**^**c**^	**Average ELISA absorbance (492 nm) of PAbs**^**e**^							
			**C57BL or B10 mouse haplotype anti-DENV-2 NS1glycoprotein PAbs**^**d**^							
**C57**				**S**	**RIII**	**G**	**BR**	**D2N**	**A**	**S(C)**
N-TERM	**+**	5-VVSWKNKELKC-15	0.87	0.92	0.78	0.71	0.67	0.70	0.68	0.18
N-TERM	**-**	15-CKLEKNKWSVV-5	0.56	0.58	0.54	0.56	0.43	0.51	0.55	0.14
LD2	**+**	25-VHTWTEQYK-33	0.67	0.54	1.21	1.32	**0.69**	0.72	1.02	0.27
LD2	**-**	33-KYQETWTHV-25	0.44	0.37	0.83	1.24	0.26	0.45	0.77	0.25
24A	**+**	61-TRLENLMWK-69	0.80	**0.77**	0.79	0.56	0.43	0.45	0.46	0.28
24A	**-**	69-KWMLNELRT-61	0.35	0.32	0.52	0.42	0.41	0.28	0.40	0.25
N'LX1	**+**	109-TELRYSWKT-117	1.23	1.41	1.01	0.97	1.14	1.16	0.96	0.27
N'LX1	**-**	117-TKWSYRLET-109	0.98	0.86	0.82	0.88	0.99	1.06	0.89	0.22
LX1	**+**	113-YSWKTWGKA-121	**0.87**	**1.08**	0.44	0.23	0.30	1.16	0.21	0.13
LX1	**-**	121-AKGWTKWSY-113	0.28	0.39	0.63	0.42	0.34	0.34	0.36	0.18
LX2/1	**+**	209-TWKIEKASF-217	0.72	0.68	1.04	1.01	0.72	0.50	0.68	0.20
LX2/1	**-**	217-FSAKEIKWT-209	0.81	1.20	1.15	1.03	1.14	0.65	0.61	0.24
LX2/2	**+**	267-PWHLGKLEM-275	0.92	0.79	1.02	1.04	0.76	0.68	0.63	0.21
LX2/2	**-**	275-MELKGLHWP-267	0.98	1.03	1.06	1.12	0.82	0.54	0.67	0.18
24C	**+**	301-TTASGKLIT-309	0.65	0.74	0.61	0.66	0.47	0.40	0.43	0.13
24C	**-**	309-TILKGSATT-301	0.41	0.47	0.51	0.56	0.42	0.39	0.48	0.12
LX2/3	**+**	331-YGMEIRPLK-339	0.65	0.71	1.24	1.01	0.69	0.81	0.62	0.26
LX2/3	**-**	339-KLPRIEMGY-331	0.61	1.11	1.21	1.16	0.51	0.66	0.59	0.20

^**a**^Name of the epitope or MAb 1G5.4-A1-C3 and/or 1C6.3 defined N’LX1, LX2/1, LX2/2 and LX2/3 peptide sequences that contained ELK/KLE-type or KELK/KLEK-type motifs.

^**b**^Synthetic peptide sequences prepared in either the natural (N: +) or reverse (R: -) orientations.

^**c**^Sequence position and amino acid sequence of the DENV-2 NS1 glycoprotein represented by synthetic peptides prepared both the natural and reverse orientations. The ELK/KLE-type and KELK/KLEK-type motifs are underlined.

^**d**^Groups of C57BL (C57) (H-2^b^) or congeneic (H-2) B10 mouse strains (B10.S (S), B10.RIII (RIII), B10.BR (BR), B10.D2N (D2N) and B10.A (A)) immunized with the native hexameric DENV-2 e/sNS1 glycoprotein (gp), or the control protein (ovalbumin) (B10.S(C): (S(C)).

^**e**^Average ELISA absorbance values of mouse PAb pools reacted with duplicate sets of synthetic peptides (standard deviation range: 0.00 (low values) to 0.04 (high values)). PAb absorbance (Abs) values which were ≥ 0.650 against the peptide prepared in the natural orientation that showed ≥ 1.50-fold or ≥ 2.50-fold RADS values (Abs against the natural (+)/reverse orientated peptide sequence), are underlined and shown in bold, respectively.

**Table 3:** Relative antibody discriminating specificity (RADS) values of MAbs and PAbs against the DENV-2 NS1 glycoprotein peptides

**MAb/PAb**^**a**^	**Relative antibody discriminating specificity (RADS) value**^**c**^								
	**Peptide/epitope on DENV-2 NS1 glycoprotein and sequence position**^**b**^								
	**N-TM**	**LD2**	**24A**	**N'LX1**	**LX1**	**LX2/1**	**LX2/2**	**24 C**	**LX2/3**
	**5-15**	**25-33**	**61-69**	**109-117**	**113-121**	**209-217**	**267-275**	**301-309**	**331-339**
**MAb**									
1H7.4	*	**16.8**	*	*	*	*	*	*	*
5H4.3	*	*	**4.2**	*	*	*	*	*	*
3D1.4	*	*	*	*	**15.0**	*	*	*	*
1G5.3	*	*	*	*	*	*	*	**12.4**	*
1C6.3	1.12	1.05	0.47	0.93	*	0.82	1.12	*	1.67
1G5.4-A1-C3	1.10	0.88	0.62	1.26	*	*	*	*	0.97
**PAb**									
C57BL	1.55	1.52	2.28	1.26	**3.11**	0.89	0.94	1.59	1.07
B10.S	1.59	*	2.40	1.64	**2.77**	0.57	0.77	1.58	0.64
B10.RIII	1.44	1.46	1.52	1.24	*	0.90	0.96	*	1.02
B10.G	1.27	1.07	*	1.10	*	0.98	0.93	1.18	0.87
B10.BR	1.56	**2.65**	*	1.15	*	0.63	0.93	*	1.35
B10.D2N	1.37	1.33	*	1.09	*	*	1.26	*	1.23
B10.A	1.24	1.33	*	*	1.08	1.11	*	*	*

^**a**^Monoclonal antibody (MAb) name or polyclonal antibody (PAb) pools from congeneic mouse strains (C57BL, B10.S, B10.RIII, B10.BR, B10.D2N and B10.A) generated against the DENV-2 NS1 glycoprotein.

^**b**^Name of epitope defined by MAb 1H7.4, 5H4.3, 3D1.4 or 1G5.3 or peptide name identified by MAb 1G5.4-A1-C3 and/or 1C6.3 through their reactions with their ELK/KLE-type or KELK/KLEK-type motifs on the DENV-2 NS1 glycoprotein with their amino acid sequence location (N-TM = peptide N-TERM).

^**c**^Relative antibody discriminating specificity (RADS) value, determined from the average ELISA absorbance values of the MAbs or mouse PAb pools against synthetic peptides prepared in the natural/reverse orientations (fold difference of absorbance against the natural orientated peptide sequence/reverse orientated peptide sequence). MAb and PAb RADS values were only determined when they had absorbance (Abs) values of ≥ 0.650 against the peptide prepared in the natural orientation. RADS values of ≥ 1.50-fold or ≥ 2.50-fold are underlined and shown in bold, respectively. Reactions that were Abs < 0.650 against the peptide sequence prepared in the natural orientation are shown by asterixes.

### MAb reactions against multiple epitopes prepared in the natural and reverse orientations using L-amino acids

**Figure 2:**
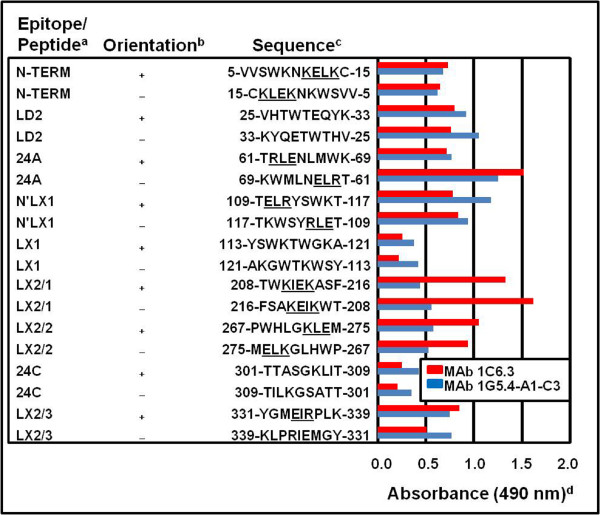
**MAb 1C6.3 and 1G5.4-A1-C3 reactions against DENV-2 NS1 glycoprotein natural and reverse orientated peptide sequences.**^a^ Peptide name. ^b^ Peptide sequence prepared in the natural (**+**) or reverse (−) orientations. ^c^Amino acid position and single letter amino acid code for peptide sequence with ELK/KLE-type and KELK/KLEK-type motifs underlined. ^d^Average ELISA absorbance (490 nm) against each duplicate synthetic peptide sequence when MAb 1C6.3 and 1G5.4-A1-C3 were diluted to 50 times their 50% end-point ELISA titers obtained against the native hexameric DENV-2 e/sNS1 glycoprotein (standard deviation range: 0.00 (low values) to 0.04 (high values)).

In contrast to the other four MAbs (Figure 1), MAbs 1C6.3 and 1G5.4-A1-C3, which defined KELK/KLEK-type and ELK/KLE-type motifs respectively, reacted with the N-TERM, 24A, N’LX1, LX2/1, LX2/2 and LX2/3 peptide sequences in the DENV-2 NS1 glycoprotein (Figure 2). Both of these MAbs also reacted with the LD2 epitope, possibly through its 31-QYK-33 sequence, but only weakly with the LX1 and 24C epitopes. MAb 1C6.3 reacted most strongly with the LX2/1 peptide sequence (Abs 1.33), while MAb 1G5.4-A1-C3 most strongly reacted with the N’LX1 peptide sequence (Abs 1.18) (partially over-lapping the LX1 epitope), when they were prepared in their natural orientation. In contrast to the high (4.2-16.8-fold) RADS values obtained for MAb 1H7.4, 5H4.3, 3D1.4 and 1G5.3 against their single target peptide sequences (Figure 1), MAbs 1C6.3 and 1G5.4-A1-C3 reacted almost equally, and sometimes more strongly, against multiple peptide sequences when they were tested in the reverse orientation (Figure 2). The highest RADS values for MAb 1C6.3 were obtained against the LX2/3 peptide sequence (1.67-fold RADS value) and for MAb 1G5.4-A1-C3 against the N’LX1 peptide sequence (1.26-fold RADS value) (Figure 2, Table 2, Table 3). They had lower RADS values against the 24A epitope and N’LX1 peptide sequence, and reacted more strongly with the reverse-orientated 24A epitope sequence, thereby giving very low 0.47-fold (MAb 1C6.3) and 0.62-fold (MAb 1G5.4-A1-C3) RADS values against it. These two MAbs therefore showed low abilities to discriminate between these natural and reverse orientated synthetic peptide sequences, despite their strong reactions against the native hexameric DENV NS1 glycoproteins in ELISA and Western blot assays (Table 1). The latter findings probably resulted from the high ELK/KLE-type and KELK/KLEK-type motif densities on these glycoproteins, which may be further increased when they were present in their native homo-hexameric forms.

### RADS values of congeneic mouse strain PAbs against epitopes on the DENV-2 NS1 glycoprotein

The ability of these RADS value measurements to dissect the target peptide sequences defined by the congeneic mouse PAbs, was also investigated. For this evaluation, RADS values of greater than or equal to 1.50-fold (low specificities) or 2.50-fold (high specificities) were determined only when absorbance (Abs) values of greater than or equal to 0.650 against the natural orientated peptide sequence were obtained.

In this study, the PAbs generated by the high-responding mouse haplotypes (I-A^b^: C57BL and I-A^s^: B10.S) had high 3.11-fold (C57BL mice) and 2.77-fold (B10.S mice) RADS values against the LX1 epitope (Table 2, Table 3). These results were consistent with their ability to react with the RLX1 peptide, and cross-react with the NS1 glycoproteins of all four DENV serotypes (Table 1). Their PAbs also displayed lower specificities for: a) the N-TERM epitope (1.55-fold (C57BL mice) and 1.59-fold (B10.S mice) RADS values), b) the 24A epitope (2.28-fold (C57BL mice) and 2.40-fold (B10.S mice) RADS values), and c) the N’LX1 peptide by the B10.S mouse PAbs (1.64-fold RADS value), which all contained KELK/KLEK-type or ELK/KLE-type motifs (Table 2, Table 3). The 24C epitope was also weakly, but specifically, identified by these PAbs (1.59 (C57BL mice) and 1.58 (B10.S mice) RADS values), while the C57BL mouse PAbs also weakly, but specifically, reacted with the LD2 epitope (1.52-fold RADS value). As observed using MAbs 1C6.3 and 1G5.4-A1-C3, these PAbs more strongly reacted with the LX2/1 peptide sequence when it was prepared in the reverse orientation but, unlike these MAbs, they more strongly reacted with the LX2/2 (C57BL and B10.S mice) and LX2/3 (B10.S mice only) peptide sequences when these were prepared in the reverse orientation.

The DENV-2 = DENV-4 ≥ DENV-1 = DENV-3 PAb reaction-profiles generated by the C57BL and B10.S mice against the NS1 glycoproteins of each DENV serotype (Table 1) could therefore be accounted for by their ability to react: i) with moderately-high RADS values against the LX1 and 24A epitopes, which were defined by dengue-complex reactive MAb 3D1.4 and the DENV-2/-4 reactive MAb 5H4.3, respectively, ii) with lower RADS values against the LD2 and 24C epitopes, which were defined by the DENV-2 and DENV-2/-4 NS1 glycoprotein reactive MAbs 1H7.4 and 1G5.3, respectively, and iii) with very low RADS values against multiple peptide sequences which contained ELK/KLE-type or KELK/KLEK-type motifs (e.g. N-TERM, LX2/1, N’LX1 (C57BL mice only), LX2/1, LX2/2 and LX2/3), that were identified by the DENV-2 complex reactive MAbs 1G5.4-A1-C3 and/or 1C6.3 (Table 2, Table 3).

In contrast, a) the DENV-2 serotype-specific NS1 glycoprotein reaction profile of the B10.BR mouse PAbs (Table 1) probably resulted from their moderately-high (2.65-fold) RADS value against the LD2 epitope (Table 2, Table 3), b) the weak DENV-2 > DENV-1 = DENV-3 = DENV-4 reaction profile of the B10.RIII mouse PAbs (Table 1) probably resulted from their low discriminating reaction (1.52-fold RADS value) against epitope 24A, and non-specific reactions against the multiple peptide sequences defined by MAb 1G5.4-A1-C3 and/or 1C6.3 (Tables 2, Tables 3), c) the very weak DENV-1 = DENV-2 = DENV-4 reaction profile of the B10.D2N mouse PAbs (Table 1) probably resulted from its weak and non-specific discriminatory reactions against the multiple peptide sequences defined by MAb 1G5.4-A1-C3 and/or 1C6.3 (Table 2, Table 3), d) the very weak DENV-2-type reaction profile of the B10.A mouse PAbs (Table 1) probably resulted from their strong reaction against the LD2 epitope (Table 3), but which was non-specific (Table 2), and e) the inability of the control B10.S mouse PAbs generated to ovalbumin to react with the DENV NS1 glycoproteins (Table 1), was consistent with their inability to react with any of these peptide sequences from the DENV-2 NS1 glycoprotein (Table 3). The DENV-2-type specific reaction profile of the B10.G mouse PAbs (Table 1) could not, however, be explained by their reactions against this panel of peptides from the DENV-2 NS1 glycoprotein (Table 2, Table 3).

The RADS methodology therefore accounted for the mouse PAb reaction profiles obtained with the native NS1 glycoproteins of each DENV serotype in almost every case. These results also confirmed that RADS values of PAbs generated against single LD2, 24A, LX1 and 24C epitopes, defined by MAb 1H7.4, 5H4.3, 3D1.4 and 1G5.3, respectively, as well as against ELK/KLE-type and KELK/KLEK-type motifs in the N-TERM, N’LX1, LX2/1, LX2/2 and LX2/3 peptide sequences (defined by MAb 1G5.4-A1-C3 and/or 1C6.3), were dependent upon the mouse H2 class II haplotypes.

### MAb 1G5.3 reactions with the 24C epitope sequence containing amino acid substitutions, deletions or cysteine-bridges

An analysis of the target 24C epitope, defined by MAb 1G5.3, was performed to identify its critical target amino acid sequence. In this study, the reaction of MAb 1G5.3 with the NS1 glycoproteins of only DENV-2 and DENV-4 was found to be dependent upon the single amino acid substitution of 303-alanine (A side-chain: -CH_3_) (299-RTTTASGKLIT-309) by valine (V side-chain: -CH-(CH_3_)_2_), present in the corresponding sequences of the DENV-1 and DENV-3 serotypes (Table 4). This MAb also showed a strong reaction against the truncated 301-TTASGKLI-307 sequence. The 303-A residue could, however, be successfully substituted by a cysteine (C side-chain: -CH_2_-SH) residue, only when it was oxidized to form an intra-cysteine bridge with another unnatural C residue (underlined) present in the same peptide (C-299-RTTTCSGKLIT-309) (intra-C-C bond). This suggested that rotation of the oxidized C residue occurred and may, therefore, be successfully replaced by smaller amino acids (e.g. glycine (G) side-chain: -H; alanine (A) side-chain: -CH_3_). MAb 1G5.3 reaction was inhibited when the 305-G (glycine: -H) was either deleted (299-RTTTASKLIT-309) or substituted either by: i) the larger hydrophobic amino acid valine (V side-chain: -CH-(CH_3_)_2_) (299-RTTTASVKLIT-309) or ii) a cysteine residue (underlined), even when it was oxidized (C299-RTTTASCKLIT-309) to form an intra C-C bond. In addition, MAb 1G5.3 was un-reactive with a peptide designed to create this epitope by the formation of an intra C-C bond (301-TTASCGGGCKLIT-309) (data not shown).

**Table 4:** MAb 1G5.3 reactions against DENV glycoprotein, HIV-1 gp41 and mammalian blood-clotting factor IX peptides

**Agent/Protein**^**a**^	**Epitope/peptide name/domain (DOM) (Status)**^**b**^	**Peptide sequence**^**c**^	**ELISA Abs**^**d**^
DENV-2/NS1	24C	299-RTTTASGKLIT-309	**3.547**
DENV-4/NS1	24C	299-RTTTASGKLVT-309	**4.324**
DENV-2/NS1	24C (303-A/V Sub)	299-RTTTVSGKLIT-309	0.236
DENV-2/NS1	24C (Rev)	309-TILKGSATTTR-299	0.232
DENV-2/NS1	24C (Core sequence)	301-TTASGKLI-308	**3.264**
DENV-2/NS1	24C (298-C Ins/303-A/C Sub/Red)	C299-RTTTCSGKLIT-309	1.032
DENV-2/NS1	24C (298-C Ins/303-A/C Sub/Ox Intra)	C299-RTTTCSGKLIT-309	**3.161**
DENV-2/NS1	24C (305-G/V Sub)	299-RTTTASVKLIT-309	0.198
DENV-2/NS1	24C (298-C Ins/305-G/C Sub/Ox Intra)	C299-RTTTASCKLIT-309	0.110
DENV-2/NS1	24C (305-G Del)	299-RTTTASKLIT-309	0.125
DENV-1/NS1	N-TERM (Red)	3-GCVINWKGRELKCG-16	0.064
DENV-2/NS1	N-TERM (Red)	3-GCVVSWKNKELKCG-16	0.111
DENV-3/NS1	N-TERM (Red)	3-GCVINWKGKELKCG-16	0.073
DENV-4/NS1	N-TERM (Red)	3-GCVVSWSGKELKCG-16	**3.602**
DENV-1-4/E	Dom III	391-WFKKGSSIG-399	0.111
DENV-1-4/E	Dom III (393-KKGSS-397 Inv)	391-WFSSGKKIG-399	0.864
DENV-4/E	Dom II (Red)	116-CAKFSCSGKITK-127	1.323
DENV-4/E	Dom II (Ox Intra)	116-CAKFSCSGKITK-127	1.951
DENV-4/E	Dom II (121-C/G Sub)	116-CAKFSGSGKITK-127	1.221
DENV-4/E	Dom III (309-C Ins/Red)	298-SYTMCSGKFSI-308C	0.122
DENV-4/E	Dom III (309-C Ins/Ox Intra)	298-SYTMCSGKFSI-308C	0.324
DENV-4/E	Dom III (309-C Ins/303-C/G Sub)	298-SYTMGSGKFSI-308C	0.342
Factor IX	Gla Dom (7-γE/E, 8-γE/G Subs)	1-YNSGKLEGFV-10	1.791
Factor IX	Gla Dom (2-N/A,7-γE/E, 8-γE/G Subs)	1-YASGKLEGFV-10	2.795
HIV-1/gp41	Gnann (Red)	598-LGIWGCSGKLICT-609	2.735
HIV-1/gp41	Gnann (Ox 603-C-608-C Intra)	598-LGIWGCSGKLICT-609	**3.673**
HIV-1/gp41	Gnann (Red)	598-LGIWGCSGKLICT-609	**3.518**
HIV-1/gp41	Gnann (Ox 603-C Inter/608-C Inter)	598-LGIWGCSGKLICT-609	**3.823**
HIV-1/gp41	Gnann (603-C and 608-C/G Subs)	598-LGIWGGSGKLIGT-609	**3.642**

^**a**^Infectious agents as dengue virus type-1 to −4 (DENV-1 to DENV-4) or human immunodeficiency virus-1 (HIV-1) and the protein as the DENV nonstructural-1 (NS1) or envelope (E) glycoprotein, HIV-1 envelope glycoprotein 41 (gp41) or the mammalian blood-clotting factor IX.

^**b**^Epitope name (e.g. the 24C or Gnann epitopes) or peptide (e.g. N-TERM) name from the DENV NS1 glycoprotein or the antigenic domain (Dom II or III) of the DENV E glycoproteins or the Gla domain (Gla Dom) of blood-clotting factor IX. The peptide status as being prepared in the reverse orientation (Rev), containing a substitution (Sub) (e.g. 303-A by V substitution = 303-A/V Sub), insertion (Ins) or deletion (Del), or having their cysteine (C) residues reduced (Red) or oxidized (Ox) to form intra-peptide (Intra) or inter-peptide (Inter) cysteine bonds.

^**c**^The amino acid sequence location, with the peptides prepared in the reverse orientation or containing substituted, inserted or inverted amino acid residues (underlined).

^**d**^Average ELISA absorbance (490 nm) against duplicate peptides when reacted at a dilution of 50 times their reciprocal 50% end-point ELISA titer obtained against the native hexameric DENV-2 NS1 glycoprotein (standard deviation range: 0.00 (low values) to 0.09 (high values)). Absorbance values above 1.000 are underlined and those above 3.000 are shown in bold.

These results showed that the reaction of MAb 1G5.3 could accept substitutions in the residues immediately adjacent to the core SGK sequence and that oxidized C residues could be substituted for the 303-A residue, but not in the core 305-G residue.

### MAb 1G5.3 reactions with epitopes on the DENV-4 NS1 and E glycoproteins

MAb 1G5.3 cross-reacted with the virion-associated envelope (E) glycoproteins (gp60 and its alternative gp55 form) of DENV-2 in immuno-blot assays, and weakly neutralized all four DENV serotypes [7]. The ability of this MAb to cross-react with the recently identified xSGKx-type motifs present on the DENV-4 NS1 and E glycoproteins was therefore investigated. Such cross-reactions may then identify a potential ‘antigenic decoy’ role of the DENV-4 NS1 glycoprotein and/or the potential of MAb 1G5.3 to generate DENV-4 antibody-enhanced replication (AER).

In this study, MAb 1G5.3 also strongly reacted with the N-TERM epitope present on the NS1 glycoprotein of DENV-4 (3-GCVVSWSGKELKCG-16), due to its unique xSGKx sequence amongst the four DENV serotypes, despite being flanked by the large tryptophan (8-W) and glutamic acid (12-E) residues (underlined) (Table 4). Interestingly, only the DENV-4 E glycoprotein, amongst the four DENV serotypes, contained surface-exposed xSGKx motifs present in the positive orientation within the corresponding neutralization epitope sequences within antigenic domain II and III, as determined from the corresponding sequences in the X-ray crystallographic analyses of the DENV-2 and DENV-3 E glycoproteins **[MMDB ID 23080 and 32273]**[63,64]. This suggested that the DENV-4 NS1 glycoprotein could contain two xSGKx epitope motifs, which could prevent PAb reactions against the surface-exposed xKGSx/xSGKx-motifs on the DENV-4 E glycoprotein. The reactions of MAb 1G5.3 with these xKGSx/xSGKx motifs were therefore tested.

MAb 1G5.3 was un-reactive with the DENV-conserved 391-WFKKGSSIG-399 sequence (Table 4), previously defined by the dengue-complex neutralizing MAb 3A8.1 (393-KKGSSIGQ/KM-410) [8]. MAb 1G5.3, however, more strongly reacted (Abs 0.864) with a peptide that contained an inverted core 393-KKGSS-397 sequence (391-WFSSGKKIG-399) which further confirmed its xSGKx motif orientation-specificity. This MAb strongly reacted with the surface-exposed 116-CAKFSCSGKITK-127 sequence (Abs 1.323) from domain II of the DENV-4 E glycoprotein, and its reaction was increased when this peptide was oxidized to form an intra-C-C bond (Abs 1.951). MAb 1G5.3 showed a slightly weaker reaction against this sequence when it contained a 121-C (C side-chain: -CH_2_-SH) by G (G side-chain: -H) substitution (116-CAKFSGSGKITK-127), to represent the rotated C residue. This suggested that substitution of the 121-C by an alanine residue (A side-chain: -CH_3_) was likely to be more suitable, as was present in the 24C epitope (299-RTTTASGKLI/VT-309) of the DENV-2 and −4 NS1 glycoproteins. MAb 1G5.3 however only weakly reacted with the 298-SYTMCSGKFSI-308C sequence, which was present in domain III of the DENV-4 E glycoprotein, in either the reduced or oxidized forms, or when the peptide contained a 302-C by G residue substitution to represent the rotated C residue. These results were probably due to the location of the large aromatic phenylalanine (306-F side-chain: -CH_2_-C_6_H_5_) residue located immediately adjacent to the SGK motif.

These results confirmed the presence of common epitopes on the DENV-4 NS1 and E glycoproteins which was due to the xSGKx ‘antigenic theme’, in addition to the KELK/KLEK-type and ELK/KLE-type ‘antigenic themes’ previously identified [6,7].

### MAb 1G5.3 cross-reaction with a critically-functional site on mammalian blood-clotting factor IXa

The ability of MAb 1G5.3 to cross-react strongly with the functional, mammalian-conserved and Ca^2+^-stabilised/activated 2-NSGKLEEFV-11 gamma-carboxyglutamic acid- (γE-) rich (Gla) domain on blood-clotting factor IXa was investigated. For this study, high-resolution x-ray crystallographic data were available for this region of the protein bound by a MAb (10C12) Fab fragment **MMDB ID 25991]**[66] or the snake (*Trimeresurus flavoviridis*) venom protein, IX/X-bp **MMDB ID 23297** and **23298]**[67]. The MAb 10C12 Fab fragment, which inhibited blood coagulation, displayed hydrophobic interactions with 6-L, 9-F and 10-V (underlined) of this sequence (1-YNSGKLγEγEFV-10). Each of the amino acids in the 3-SGKL-6 core target sequence defined by MAb 1G5.3 were highly exposed, while one COO^-^ group of the 7-γE (side-chain: -CH_2_-CH-(COO^-^)_2_) residue bound a Ca^2+^ ion, the other COO^-^ group of its side-chain was available for interaction with MAb 1G5.3. The side-chain of its 8-γE residue was instead buried, due to the interaction of both of its COO^-^ groups with Ca^2+^ ions. As such, these amino acids should be suitably replaced by a normal glutamic acid (7-E side-chain: -CH_2_-CH_2_-COO^-^) residue, and a glycine (8-G side-chain: -H) residue. The tyrosine (1-Y) side-chain was exposed, but the side-chain of the critical adjacent asparagine (2-N side-chain: -CH_2_-C(NH_2_) = O) residue was rotated to bind a Ca^2+^ ion, thereby being unavailable for full interaction with MAb 1G5.3. Interestingly, this same molecular structure of the 1-YNSGKLγEγEFV-10 sequence was also identified when it contained bound Ca^2+^ or Ca^2+^ and Mg^2+^ ions, and the IX/X-bp snake venom protein [61]. As a result, this 2-N residue was likely to be more suitably substituted by an alanine or glycine (A side-chain: -CH_3_; G side-chain: -H) residue. These observations were consistent with the strong binding (Abs 1.791) of MAb 1G5.3 to this peptide sequence when it contained 7-E and 8-G residues (1-YNSGKLEGFV-10), and even stronger binding (Abs 2.795) when this peptide also contained a 2-N by A substitution (1-YASGKLEGFV-10) (Table 4). These results may, therefore, account for the ability of MAb 1G5.3 to generate dramatic intra-peritoneal haemorrhage in mice [23].

### Reaction of MAb 1G5.3 with an immuno-dominant epitope on human immunodeficiency virus-1 (HIV-1) glycoprotein 41

HIV-1-infected patients, particularly those with AIDS, generated PAb responses against the immuno-dominant 599-GIWGCSGKLIC-609 (Gnann epitope) sequence on the 41 kDa glycoprotein (gp41) of HIV-1, which could be faithfully represented as an intra- C-C bridged synthetic peptide [57,58]. The X-ray crystallographic structure of this peptide (600-IWGCSGKLICTTA-612) had also been determined **[MMDB ID: 73687]**[65]. MAb 1G5.3 was tested for its ability to react with this determinant when it was reduced (unnatural), or contained either an inter- (unnatural) or intra- (natural) cysteine bridge (C-C), or C by G substitutions (unnatural).

In this study, MAb 1G5.3 reacted with this epitope in the reduced form (Abs 2.74), but its reaction with it was dramatically increased when both C (603-C and 608-C) residues were oxidized to form either the natural intra-C-C (natural) peptide loop (Abs 3.67), or inter-peptide (unnatural) C-C bonds (Abs 3.82) (Table 4). The strong reaction of MAb 1G5.3 with the latter peptide, even before it was oxidized (pre-oxidation: Abs 3.52; post-oxidation: Abs 3.82), probably resulted from the partial rotation of the 603-C residue due to the presence of the un-cleaved (I_2_-sensitive) hydrophobic S-acetamidomethyl (S-Acm: -S-CH_2_-NH-CO-CH_3_) protective group. Similar strong MAb 1G5.3 reactions (Abs 3.64) were observed when this peptide sequence was prepared with 603-C and 609-C by G substitutions to represent the rotated C side-chains. Thus, despite MAb 1G5.3 being generated against the DENV-2 NS1 glycoprotein, it showed a similarly strong reaction against an immuno-dominant epitope on HIV-1 gp41 (Abs 3.55 versus 3.52-3.82) which was previously shown to generate HIV-1 AER via complement receptor- [59] or, complement-independent Fcγ-receptor- [60] mediated mechanisms. MAb 1G5.3 is therefore also likely to generate AER of this heterologous infectious agent.

## Discussion

The main findings from this study were that: a) MAb and PAb RADS values, and their reactions against large numbers of peptides that contained amino acid inversions, deletions, substitutions or intra-/inter-C-C bridges, could be concurrently assessed in the convenient 96-well ELISA format, b) the measurements of RADS values allowed discriminating MAb and PAb reactions to be distinguished from non-discriminating ones using a large number of peptide sequences to account for their reaction patterns against natural NS1 glycoproteins of all four DENV serotypes, c) common epitopes were present on the DENV-2 and −4 NS1 glycoproteins and on a critical phospholipid-binding site on mammalian blood-clotting factor IXa which may account for the ability of MAb 1G5.3 to generate severe haemorrhage *in vivo*, d) common motifs on the DENV-4 NS1 and E glycoproteins were identified by their xSGKx ‘antigenic theme’ and the reaction of MAb 1G5.3 with the 116–127 peptide sequence in domain II of the DENV-4 E glycoprotein was increased when it contained an intra-C-C bridge or amino acid substitutions mimicking more faithfully its natural X-ray crystallograph, e) this was the first report that identified a common determinant on a DENV protein and another infectious agent (HIV-1 gp41) which could generate antibody-enhanced replication (AER), and f) the methodology described may be useful to identify patients’ PAb reactions that are involved in the pathogenesis of DENV, or other infectious and non-infectious diseases.

### Peptide methodology

Importantly, only natural L-amino acid residues were used to prepare the peptides in both the natural and reverse orientations for the RADS value determinations, rather than using D-amino acid residues as used previously in the preparation of ‘retro-inverso’ peptides [68].

In this study, all of the peptides except the RLX1 peptide, were covalently attached to uniformly-loaded aliphatic spacer molecules on ‘gears/arrowheads’ so that each amino acid side-chain was exposed for MAb/PAb binding. This improved technology resulted in much lower variations in the peptide concentrations and MAb/PAb reactions [6,7], as was previously observed with the ‘pin’ technology [23,44]. This method was therefore unlike that in which peptides are adsorbed onto solid-phase ELISA plates where some of the amino acid side-chains, particularly of short peptide sequences, would be unavailable for MAb/PAb binding. In the case of the RLX1 peptide, the longer (19-mer) peptide length, and dimerization via its carboxyl-terminal cysteine (C) residue, as demonstrated for the AFLX1 peptide [6,23], allowed all of the essential amino acid side-chains of epitope LX1 to be exposed for MAb and PAb reactions [6,23]. It is possible that this method could have increased the accessibility to antibodies thereby resulting in an increased detection of cross-reactions with heterologous peptide sequences. The cross-reactions of these MAbs and mouse PAbs from all congeneic (class II) mouse strains used in this study had previously been confirmed by their reactions with epitopes and critical functional sites in multiple proteins of human platelets, endothelial cells and blood-clotting factor I (fibrinogen) [6,23] (see below).

### Relative antibody discriminating versus non-discriminating MAb reactions against DENV proteins

Since MAb 3D1.4 showed a highly specific 15.00-fold RADS value against its DENV complex-conserved LX1 epitope, it was useful for detecting the s/eNS1 glycoproteins in DENV-infected patients’ blood/serum samples using simple, inexpensive and sensitive DIS and N-DIS dot-blot assays [69], as well as some expensive commercially-available DENV diagnostic assays [70,71].

Antigenic ‘poly-specificity’ of PAbs and MAbs has frequently been reported [9-14]. Thus, despite MAb 1G5.3 having a high RADS value against the target 24C epitope on the NS1 glycoproteins of DENV-2/-4, it also reacted strongly with other natural-orientated core target xSGKx sequence on the DENV-4 E and NS1 glycoproteins, HIV gp41, as well as with a critical site on mammalian blood-clotting factor IXa.

The DENV-2 NS1 and E glycoproteins were shown to have antigenically co-evolved by immuno-blot analyses of these glycoproteins from different DENV-2 strains using MAbs [42]. These results were further supported by phylogenetic analyses of the genes encoding the E and NS1 glycoproteins of all DENV serotypes [43]. In this study, MAb 1G5.3 was used to identify xSGKx motifs that were uniquely present in the NS1 glycoprotein N-TERM peptide sequence and 116–127 peptide sequence of the DENV-4 E glycoprotein (domain II), which supported these earlier observations. MAb 1G5.3 was previously shown to: i) weakly bind to the flavivirus-conserved neutralization fusion-sequence on the E glycoprotein, ii) weakly neutralize all DENV serotypes, iii) generate intraperitoneal haemorrhage in mice, and iv) generate a dramatic and lethal AER/AED of a DENV-2 strain [6,45]. Further studies are required to assess the potential ‘decoy’ effect of the large concentrations of the NS1 glycoprotein that are secreted con-currently with virions from DENV-4-infected cells, and whether this glycoprotein could inhibit or reduce the ability of MAb 1G5.3 to react with this epitope on the DENV-4 E glycoprotein, and thereby generating its AER resulting in AED.

Many KELK/KLEK-type and ELK/KLE-type motifs, defined by MAbs 1C6.3 and 1G5.4-A1-C3, were located in or immediately flanking the sequential epitopes identified by four other MAbs (e.g. the 24A and N’LX1 epitopes), and which were immuno-dominant in both mice and humans [6,23]. The N’TERM peptide sequence, 1-DSGCVVSWKNKELKC-15 [6,23], was reported to generate predominantly an immuno-dominant IgM response in 45% of humans with primary or secondary DENV infections, which could be detected as early as day 2 after the onset of febrile illness in some patients [41]. This peptide sequence also contained a KELK motif (underlined), which was confirmed to be surface-exposed and antigenic in the DENV-2 E glycoprotein (156-GKHGKEI/VKIT-165) [6,7]. RADS value analysis would therefore be useful for elucidating whether these human IgM PAbs were generated against this epitope *per se*, or whether these IgM PAbs were generated against similar epitope sequences on the more immunogenic DENV-2 E glycoprotein. In other studies, DENV NS1 glycoprotein-reactive IgG PAbs were only detected during the acute-phase of secondary DENV infections [41], when most DHF/DSS cases occur [26,28]. The IgG PAbs generated by three (C57BL, B10.S and B10.BR) of the seven (3/7: 42.9%) congeneic mouse strains tested, showed only weak specificities (i.e. low RADS values) against the N-TERM epitope. All of the congeneic mouse strains instead generated the highest IgG absorbance values against the N’LX1peptide sequence, but were only weakly discriminatory (low RADS values). The highest RADS values for the PAbs were obtained for PAbs to the LX1 and 24A epitopes from the C57BL and B10.S mice, and against the LD2 epitope by the PAbs from the B10.BR mice, and accounted for their reaction profiles against the NS1 glycoproteins of each DENV serotype. These results therefore showed that the immuno-dominance of particular epitopes was dependent on their class II haplotypes. The X-ray crystallographic structure of the DENV NS1 glycoprotein has not been determined to date. The results from this study however strongly suggest that immunodominant antibody responses were generated against the ELK/KLE-type/KELK/KLEK-type motifs due to their high prevalence within sequential epitopes(e.g. the N-TERM and 24A epitopes) or immediately flanking other epitopes (e.g. the N'LX1 peptide partially overlapping the LX1 epitope) that were identified with moderately-high RADS values by both MAbs and PAbs. The similar reactions of both MAbs 1G5.4-A1-C3 and 1C6.3 with ELK/KLE-type and KELK/KLEK-type motifs in DENV NS1 and E glycoprotein sequences when presented in both natural and reverse orientations suggested that these reactions were strongly dependent on electrostatic interactions, while the central residue may contribute to hydrophobic associations. The importance of ELK/KLE-type motif density was supported by the finding that these ELK/KLE-type motifs were more numerous and/or antigenic in surface-exposed regions of the E glycoproteins from virulent (DSS-associated) DENV-2 and DENV-3 strains, while MAb 1G5.4-A1-C3 weakly neutralized these DENV strains [7]. Importantly, the DENV E glycoproteins exist as homodimers, which form homotrimers upon target cell fusion [63,64] which would increase the reactions of MAbs and PAbs due to their ELK/KLE-type/KELK/KLEK-type densities. MAb 1G5.4-A1-C3 is therefore also more likely to generate the AER of these more virulent DENV strains. These results also agree with those obtained in several studies that demonstrated strong direct correlations between antigenicity and immunogenicity and epitope densities [46-49].

### Reaction of MAb 1G5.3 with mammalian blood-clotting factor IXa

During the critical stage of disease (DHF/DSS), usually day 4–5 after the onset of the febrile phase DHF/DSS (DHF grade I to IV), patients show thrombocytopenia, haemoconcentration (increased haematocrit percentages) and may also display spontaneous bleeding (DHF grades II-IV) [72,73]. Early evidence of bleeding (conjunctival injection and veni-puncture bleeding), displayed during the first 72 hours after the onset of fever in the absence of thrombocytopenia, amongst other clinical criteria, definitively identified DENV-infected patients who subsequently developed DHF/DSS [74]. MAb 1G5.3 displayed a strong ‘cross-reaction’ with the critical phospholipid-binding site (Gla domain (ω-loop)) of mammalian blood-clotting factor IXa, which may have accounted for its ability to generate intra-peritoneal bleeding in mice [23]. This was supported by X-ray crystallographic analyses of the critical target sequence on factor IXa bound by MAb 10C12 or the IX/X-bp snake venom protein, which both inhibited blood-coagulation [61,66,67]. Interestingly, patients with haemophilia type B caused by a single 2-asparagine (2-N) by tyrosine (Y) substitution (1-YYSGKLγEγEFV-10) had a 99% reduction in their factor IXa function [75]. Haemophilia type B is displayed by severe and often spontaneous haemorrhagic disease (Christmas disease), similar to that observed in some patients with severe DENV disease (DHF grades II to IV) [72,73]. These findings, therefore, confirmed the essential role of: i) factor IXa in maintaining normal haemostasis, despite its very low blood concentration (44–88 nM: mean 67 nM = 0.038 μg/ml) [76], and ii) the 2-N residue binding to a Ca^2+^ ion thereby causing its rotation, and which would result in increased MAb 1G5.3 binding. Reduced functions of factor IXa were however only found in 17% of patients with DHF/DSS, while 50% and 100% showed reduced factor VIIa and factor I (fibrinogen) functions/concentrations, respectively [77]. Such findings of factor IXa concentrations in DHF/DSS patients may, therefore, be consistent with the observation that only mice that possessed the highest responding class II haplotypes (C57BL: I-A^b^ and B10.S: I-A^s^) (2/7: 29%) generated PAbs with relatively low 1.58- and 1.59-fold RADS values against the 24C epitope in this study.

While an initial study showed that MAb 1G5.4-A1-C3 bound to the corresponding Gla domain peptide sequence of factor VIIa (1-ANAFLγEγELRP-10) [23], the more recent X-ray crystallographic analysis of this region of factor VIIa **[MMDB ID 70357]**[78] showed that both carboxylic acid groups of 5- and 6-γE residues were bound to Ca^2+^ ions. MAb 1G5.4-A1-C3 would therefore be unable to bind the target 6-γELR-9 (ELK-motif) sequence on activated factor VIIa. The reduced function of factor VIIa in DHF/DSS patients may therefore result from either other biochemical factors, or from their PAb reactions against other epitopes on this molecule. In contrast, immuno-dominant PAb responses were generated against these ELK/KLE-type motifs in all congeneic mouse strains tested when these animals were either immunized with the DENV-2 NS1 glycoprotein or infected with live DENV-2, and all of them reacted with human platelets and fibrinogen [6,23]. These ELK/KLE-type motifs were identified by MAb 1G5.4-A1-C3 in multiple surface-exposed regions of the human fibrinogen (α- and γ-chains) which were functionally active sites (e.g. xRGDx-motifs and the xRLDGSx-motif) [23]. PAb responses against ELK/KLE-type motifs were higher in DSS patients versus DF patients, and DSS patients sera showed the same high reactions against multiple ELK/KLE-type motifs in the DENV-2 NS1 glycoprotein as MAb 1G5.4-A1-C3 and sera from high responding congeneic (H-2^b^ and H-2^s^) mouse strains immunized with this glycoprotein [6,23]. This is consistent with the high concentrations of fibrinogen that have been identified in DSS patients’ circulating immune complexes (CICs) [45,79] and with the fact that single domain anti-fibrinogen MAbs generated against the DENV-2 NS1 glycoprotein prolonged thrombin times in blood-coagulation assays [80]. It is likely that such PAb reactions may account for the consistently reduced detection of fibrinogen and fibrinogen-degradation products in DHF/DSS patients blood samples [73,79,81]. Further studies are therefore required to confirm whether DSS patients’ CICs also contain blood-clotting factor VIIa and/or IXa.

### Cross-reaction of MAb 1G5.3 with an important epitope on another infectious agent

In this study, MAb 1G5.3 strongly reacted with the immuno-dominant epitope on HIV-1 gp41. Initial studies suggest that PAbs from HIV-1-infected patients also react with epitopes on the DENV E glycoproteins (data not shown). This was the first identification of common determinants on DENV and another infectious agent, and such poly-reactive antibodies may generate AER of these heterologous viruses. Importantly, the ability of PAbs generated against HIV-1 gp41 or MAb 1G5.3 to generate DENV-4 AER/AED can be further tested under physiological conditions *in vivo*, using a recently developed DENV AER/AED model [24].

### Potential design of improved peptide sequences

The oxidation of cysteine residues to form natural loops was essential for optimal antigenicity of a protective epitope on the hepatitis B virus [82] and a diagnostic peptide on HIV-1 gp41 [57,58]. The addition and oxidation of cysteine residues flanking a loop-configured epitope defined by an anti-DENV E glycoprotein MAb which neutralized all DENV serotypes [8] is also likely to increase its antigenicity. Increased antigenicity was observed in this study by the substitution of the C by A or G residues (C side-chain: -CH3; G side-chain: -H) corresponding to the rotation of the oxidized C residues in the DENV-4 E glycoprotein. Previously, a C by G amino acid substitution in the conserved flavivirus E glycoprotein fusion sequence (101-WGNGCGLFG-109) was also tested [8]. In this previous study, this single 105-C by G (G side-chain: -H) substitution dramatically increased the binding of flavivirus group (MAb 4G2 and 2C5.1) and sub-group (MAb 2F2.1) reactive neutralizing MAbs, which could also be gauged by their increased RADS values from 1.64, 1.67 and 1.31 to 2.60, 2.57 and 2.33, respectively against these peptide sequences [8]. These results were consistent with the rotation of the 105-C residue flanked by the other critical 104-G, 106-G and 107-L residues required for MAb 4G2 binding [83], and the weak ability (65% inhibition at 1 mM) of a synthetic peptide that contained a 105-C by S (serine) (side chain: -CH_2_-OH) to block a neutralizing MAb binding to its target fusion sequence on DENV particles [84]. In addition, new improved hybrid target sequences, defined by protective anti-DENV prM and E MAbs have also been designed [62]. The substitution of the critical 2-N by G or A, and 7γE by E and 8γE by G are also likely to more closely mimic the activated phospho-lipid binding site of activated (Ca^2+^-stabilized) human blood-clotting factor IX (IXa), as shown in the structure of this critical site determined by X-ray crystallography [66,67].

### Potential applications for DHF/DSS pathogenesis studies

We described simple, definitive prognostic clinical criteria that could specifically identify DENV-infected patients, during the early acute phase of disease (≤ 72 hours after the onset of fever), who will subsequently develop DHF/DSS versus DF or had another febrile illness [74]. Early acute phase PAb samples from DENV-infected patients against a synthetic peptide identified those patients who subsequently developed DSS, but not DHF or DF [45]. A panel of MAbs has been tested for their ability to generate DENV AER/AED *in vivo* at high concentrations using our mouse model [24], to prevent confusion with the ability of neutralizing MAbs to generate DENV AER when diluted beyond their effective neutralizing concentrations [26]. It is therefore essential for a tetravalent DENV vaccine to generate adequate and sustainable levels of neutralizing antibodies against each of the four DENV serotypes [37]. Of further concern is that such a vaccine may also place infants (mean age: 6-months), who have low and broadly DENV cross-reactive IgG1 antibodies during their weaning stage, at high risk for developing DHF/DSS in primary DENV infections as was shown in Cuba, Singapore [85] and Viet Nam [86,87].

RADS values, obtained against the peptide sequences on the DENV E glycoproteins and auto-antigens, defined by those MAbs that generated DENV AER/AED in our lethal *in vivo* DSS model [24] will be determined and assessed in the future for their potential prognostic values for DHF/DSS patients to support the definitive clinical criteria already identified [74].

## Conclusions

In conclusion, the RADS value methodology, together with amino acid deletion, substitution and inter- and intra-C-C bridge formation analyses, that were evaluated using multiple synthetic peptides covalently attached by their carboxyl-termini in the standard 96-well ELISA format, was very useful to gauge the discriminating reactions against epitopes that were recognized by PAbs or MAbs using synthetic peptides. The methodology described was therefore useful to: a) confirm the occurrence of cross-reactions between epitopes by their RADS values in: i) the same viral protein (e.g. DENV-4 NS1 glycoprotein), ii) another DENV glycoprotein (e.g. the DENV-4 E glycoprotein), iii) another infectious agent (e.g. HIV-1), and iv) a mammalian glycoprotein (e.g. blood-clotting factor IXa), and could be used to design more antigenic peptide sequences. In the latter case, amino acid substitutions in synthetic peptide sequences can be used to represent rotated amino acids in the DENV-4 (116-CAKFSCSGKITK-127) E glycoprotein, and the activated form of the human blood-clotting factor IX (1-YNSGKLγEγEFV-10), in order to mimic their native conformations. These findings are important for understanding the pathogenesis of DHF/DSS caused by either auto-immune reactions [6,23] or DENV AER, which can be confirmed *in vivo* by their abilities to generate severe, lethal multi-organ disease syndrome (MODS) [24], and may lead to the design of suitable prognostic peptides for DHF/DSS patients. Importantly, the methodology described will also be useful for assessing discriminating MAb or PAb reaction specificities against epitopes on proteins of any pathogen, allergen or auto-antigen.

## Competing interests

The author declares that no conflicts of interest exist.

## Author’s contributions

The author designed all of the experiments, prepared all of the MAbs, PAbs and peptides described, performed all of the experiments, analyzed the data, prepared the figures and tables, and wrote the paper.
